# Enhanced parasympathetic cholinergic activity with galantamine inhibited lipid-induced oxidative stress in obese African Americans

**DOI:** 10.1186/s10020-022-00486-5

**Published:** 2022-06-03

**Authors:** Dena Parsa, Luul A. Aden, Ashley Pitzer, Tan Ding, Chang Yu, Andre Diedrich, Ginger L. Milne, Annet Kirabo, Cyndya A. Shibao

**Affiliations:** 1 Division of Clinical Pharmacology, Department of Medicine, Vanderbilt University Medical Center, 506 Robinson Research Building, Nashville, TN 37232 USA; 2 Department of Biostatistics, Vanderbilt University Medical Center, Nashville, TN USA; 3 Department of Molecular Physiology and Biophysics, Vanderbilt University, Nashville, TN USA; 4 Department of Biomedical Engineering, Vanderbilt University, Nashville, TN USA

**Keywords:** Oxidative stress, African American, Lipids, F_2_-isoprostanes, Central acetylcholinesterase inhibitor

## Abstract

**Background:**

African Americans (AAs) are disproportionately affected by cardiovascular disease (CVD), they are 20% more likely to die from CVD than whites, chronic exposure to inflammation and oxidative stress contributes to CVD. In previous studies, enhancing parasympathetic cholinergic activity has been shown to decrease inflammation. Considering that AAs have decreased parasympathetic activity compared to whites, we hypothesize that stimulating it with a central acetylcholinesterase (AChE) inhibitor, galantamine, would prevent lipid-induced oxidative stress.

**Objective:**

To test the hypothesis that acute dose of galantamine, an AChE inhibitor, decreases lipid-induced oxidative stress in obese AAs.

**Methods:**

Proof-of-concept, double-blind, randomized, placebo-controlled, crossover study that tested the effect of a single dose of 16 mg of galantamine versus placebo on lipid-induced oxidative stress in obese AAs. Subjects were studied on two separate days, one week apart. In each study day, 16 mg or matching placebo was administered before 20% intralipids infusion at doses of 0.8 mL/m2/min with heparin at doses of 200 U/h for 4 h. Outcomes were assessed at baseline, 2 and 4 h during the infusion.

**Main outcome measures:**

Changes in F_2_-isoprostane (F_2_-IsoPs), marker of oxidative stress, measured in peripheral blood mononuclear cells (PBMC) and in plasma at baseline, 2, and 4-h post-lipid infusion. Secondary outcomes include changes in inflammatory cytokines (IL-6, TNF alpha).

**Results:**

A total of 32 obese AA women were screened and fourteen completed the study (age 37.8 ± 10.70 years old, BMI 38.7 ± 3.40 kg/m^2^). Compared to placebo, 16 mg of galantamine significantly inhibited the increase in F_2_-IsoPs in PBMC (0.007 ± 0.008 vs. − 0.002 ± 0.006 ng/sample, P = 0.016), and plasma (0.01 ± 0.02 vs. − 0.003 ± 0.01 ng/mL, P = 0.023). Galantamine also decreased IL-6 (11.4 ± 18.45 vs. 7.7 ± 15.10 pg/mL, P = 0.021) and TNFα levels (18.6 ± 16.33 vs. 12.9 ± 6.16 pg/mL, P = 0.021, 4-h post lipid infusion) compared with placebo. These changes were associated with an increased plasma acetylcholine levels induced by galantamine (50.5 ± 10.49 vs. 43.6 ± 13.38 during placebo pg/uL, P = 0.025).

**Conclusions:**

In this pilot, proof-of-concept study, enhancing parasympathetic nervous system (PNS) cholinergic activity with galantamine inhibited lipid-induced oxidative stress and inflammation induced by lipid infusion in obese AAs.

*Trial registration:* ClinicalTrials.gov identifiers NCT02365285.

## Background

African Americans (AA) account for ~ 13% of the US population; they are at a disproportionately affected by cardiovascular disease (CVD), which is estimated to contribute to more than 2 million AA lives lost in the past decade (Gillespie et al. 2009). The high prevalence of CVD in AAs is, in part, explained by the disproportionate presence of traditional risk factors such as obesity (55%), hypertension (44%), and type 2 diabetes mellitus (T2DM) in this population, which affects particularly AA women (Carnethon et al. 2017; Hales et al. 2017). However, non-traditional CV risk factors such as chronic sub-clinical inflammation and oxidative stress also contributes to the pathogenesis of CVD (Roger et al. 2012). In this context, previous studies found that AAs have increased activation of nicotinamide adenine dinucleotide phosphate (NADPH) oxidase, which is capable of producing large amounts of reactive oxygen species (ROS) and superoxide in peripheral blood mononuclear cells (PBMC) (Deo et al. 2015). Higher protein expression of NADPH oxidase subunit p47phox as well as higher IL-6 levels has been reported in AA women than white controls (Feairheller et al. 2011). In addition, lipid infusion, which enrich the production of ROS, increased plasma levels of F_2_-isoprostane (F_2_-IsoPs), a biomarker of endogenous oxidative stress, by two-fold in AAs compared with whites (Lopes et al. 2003). In this regard, previous work by Tracey and collaborators (Borovikova et al. 2000) showed that the PNS is an important regulator of inflammation and oxidative stress; its stimulation confers protection against heightened oxidative stress states induced by fulminant hepatitis or ricin poisoning (Mabley et al. 2009; Abdel-Salam et al. 2013). Likewise, central PNS cholinergic stimulation with galantamine, a central acetylcholinesterase (AchE) inhibitor showed neuro protection in rat hippocampal slices stressed with oxygen and glucose deprivation through NADPH oxidase inhibition (Lorrio et al. 2007) Altogether, these studies led us to hypothesize that galantamine could reduce lipid-induced oxidative stress and inflammation, measured with F_2_-IsoPs and cytokine levels in obese AAs.

## Methods

### Study design

**Fig. 1:**
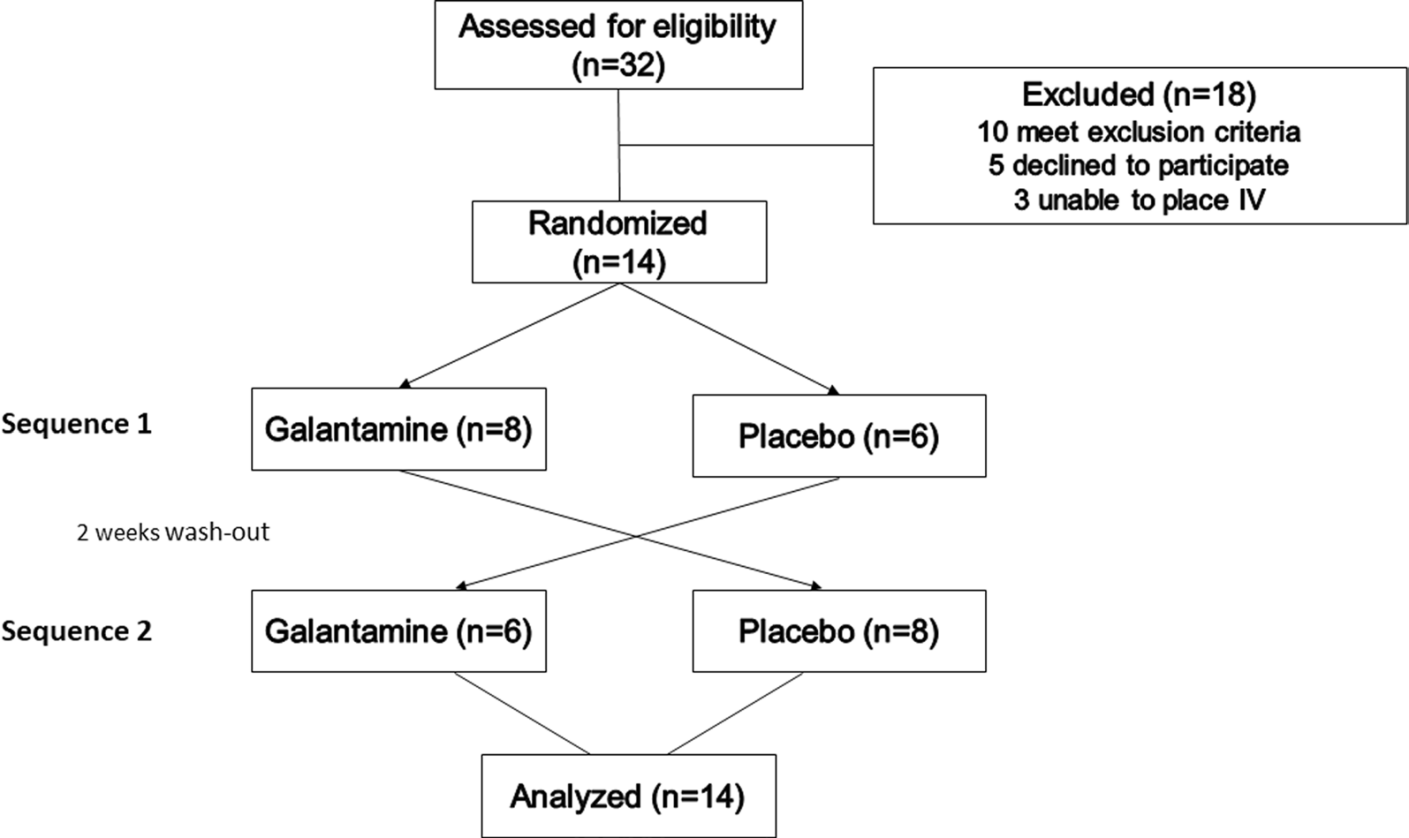
Enrollment flow. The study was a double-blind, randomized, placebo-controlled, 2 × 2 crossover design. A total of 32 subjects were screened, 18 were excluded. Fourteen were randomly assigned to sequence 1 (16 mg galantamine followed by placebo) or sequence 2 (placebo followed by 16 mg of galantamine)

The study was conducted at the Vanderbilt Clinical Research Center (CRC). The study design was a randomized, double-blind, placebo-controlled, 2 × 2 crossover trial that compared the effect of 16 mg galantamine versus placebo on lipid-induced oxidative stress. Participants were assigned randomly to treatment sequences (galantamine followed by placebo [sequence one], or vice versa [sequence two]). In between treatments, there was a 2-week washout period. Both, study team and study subjects were blinded to the study sequence (Fig. 1). All studies adhered to the principles of the Declaration of Helsinki and Title 45, U.S. Code of Federal Regulations, Part 46, Protection of Human Subjects. The Vanderbilt Institutional Review Board approved these studies, and they were conducted in accordance with institutional guidelines. All subjects provided informed consent; the studies were registered on ClinicalTrials.gov identifiers NCT02365285.

### Study population

Eligibility criteria included obese women (as defined as Body Mass Index (BMI) between 30 and 45 kg/m^2^), aged 18 to 60 years old, and AA; race was self-defined, but only subjects who reported both parents of the same race were included. We excluded pregnant or breastfeeding women, individuals diagnosed with type 2 diabetes mellitus, hypertension or any cardiovascular disease, impaired renal function (glomerular filtration rate, GFR < 60%), impaired hepatic function (abnormal liver function test), or had a history of alcohol or drug abuse. Subjects were also excluded if they used potent inhibitors of Cytochrome P450 (CYP) 3A4, CYP 2D6, AchE inhibitors such as pyridostigmine, bethanechol, or had a significant change in weight ≥ 5% in the previous three months.

After informed consent was obtained, the participants were invited to come to the CRC for a screening visit that consisted of a complete history, physical exam, laboratory assessment (complete blood count, fasting lipid profile, comprehensive metabolic panel, urine beta-hCG **(**human chorionic gonadotropin), and an electrocardiogram (ECG). In a separate visit, subjects returned to the CRC to complete a 75 g oral glucose tolerance test and body composition measured with dual-energy X-ray absorptiometry (Lunar IDXA, GE Healthcare, CT, USA).

Subjects who met the eligibility criteria were admitted to the CRC for their first study day (treatment sequence one, see Fig. 1). Before admission, they were asked to collect 24-h urine for sodium, creatinine, and F_2_-IsoPs measurements. An intravenous catheter was placed in one arm for blood sampling and another in the contralateral arm for lipid and heparin infusion. Blood samples were collected from non-Esterified free Fatty Acids (NEFA), triglycerides, plasma F_2_-IsoPs, prostaglandin F2a (PGF2a), inflammatory cytokines (TNFa, IL-6, IL-10), and acetylcholine levels. PBMC were also isolated for measurements of F_2_-IsoPs. The study nurse administered the blinded medication one hour prior to a continuous infusion of 20% Intralipids® (Baxter Healthcare Crop. Glendale, CA) at a rate of 0.8 mL/m^2^/min as previously described (Lopes et al. 2003). In addition, the study subject received a heparin bolus of 1000 units followed by a heparin infusion at a rate of 200U/h for 4-h to activate the endothelial lipoprotein lipase and accelerate the hydrolysis of fatty acids from the glycerol backbone of the triglycerides. Intermittent blood pressures and ECG were measured using the VITAL-GUARD 450c monitor (Ivy Biomedical Systems, Brandford, CT, USA). Contrast-enhanced ultrasonography (CEU) was used to measure changes in microvascular blood volume in the skeletal muscle. All measurements were repeated at 2 and 4-h post lipid infusion. We only obtained the CEU measurements and urine samples for sodium, creatinine, and F_2_-IsoPs assessments at 4-h. Subjects washed out for two weeks and the subject completed sequence two and underwent all the procedures outlined previously.

### Randomization

Subjects were randomly assigned to the treatment sequences using a permuted-block randomization algorithm. The Vanderbilt Investigational Pharmacy was responsible for the randomization, storage, preparation, and labeling of all investigational agents (Intralipids®, heparin, and blinded study drug) and for maintaining accurate drug storage and dispensing logs.

### Intervention

Our intervention was 16 mg of galantamine hydrobromide (Razadyne®) versus matching placebo. Galantamine is a competitive central AchE inhibitor that increases the availability of acetylcholine. This drug is FDA-approved for the treatment of Alzheimer’s dementia. We chose a dose of 16 mg p.o. based on pharmacokinetic studies in normal volunteers and patients with dementia of Alzheimer’s type. The 16 mg dose exhibits linear pharmacokinetics after an oral administration; the oral bioavailability is about 90%^9^ and the maximum concentration is achieved at 1-h post-administration with a short half-life of 7-h. Our measurements at 2 and 4-h coincide with the peak concentration of galantamine after administration.

### Endpoints

The primary endpoint of this study was the change in plasma F_2_-IsoPs with lipid infusion (ΔISO) during placebo versus 16 mg of galantamine.

Secondary endpoints were the changes in F_2_-IsoPs in PBMC and inflammatory cytokines post-lipid infusion during placebo versus galantamine.

### Clinical chemistry

The blood samples were collected in chilled Ethylenediaminetetraacetic acid (EDTA) tubes and were immediately centrifuged to separate the plasma, and stored at − 80 °C. For serum, the blood was clotted at room temperature for 20 min and centrifuged; the serum was removed and stored at − 80 °C. Plasma glucose was measured at the bedside with a glucose analyzer (YSI Life Sciences, Yellow Springs, OH). Plasma insulin concentrations were determined by radioimmunoassay (Millipore, St. Charles, MO).

Tetrahydrolipstatin was added to the NEFA collection tube to prevent in vitro lipolysis (Nordestgaard and Varbo 2014). Serum NEFA were measured as previously published (Krebs et al. 2000) and Triglycerides (Roche Diagnostics, Indianapolis, IN) by enzymatic colorimetry Cliniqa (Nordestgaard and Varbo 2014) for microtiter plates.

Inflammatory cytokines (TNFa, IL-6, IL-10) were measured with Multiplex Luminex technology using x-map MagPix system (Millipex map human cytokine/chemokine magnetic bead panel, Millipore Sigma, MS, US). The protocol for isolation of mononuclear cells from peripheral human whole blood was adapted from the Ficoll-Paque PLUS instruction 71–7167-00 AG (GE Health Care) document (Milne et al. 2015).

### F_2_-isoprostane assessment in PBMC

1 mL cell culture media was added to 1.0 ng of [^2^H_4_]-15-F_2t_-IsoP ([^2^H_4_]-8-iso- PGF2a; Cayman Chemical, Ann Arbor, MI) internal standard. The solution was adjusted to pH 3 with 1 N HCl. The sample was then applied to a C-18 Sep-Pak cartridge that has been pre-washed with 5 ml methanol and 5 ml 0.01 N HCl. The cartridge was then washed with 10 ml 0.01 N HCl, followed by 10 ml heptane, and compounds were then eluted with 10 ml ethyl acetate: heptane (50:50, v/v). The eluate was applied to a silica Sep-Pak cartridge prewashed with ethyl acetate (5 ml) rinsed with 5 ml ethyl acetate and compounds eluted with 5 ml ethyl acetate: methanol (50:50, v/v). The eluate was dried under nitrogen. Compounds were converted to the pentafluorobenzyl (PFB) esters by the addition of 40 μl of a 10% solution of pentafluorobenzyl bromide in acetonitrile and 20 μl of a solution of 10% diisopropylethanolamine in acetonitrile and allowed to incubate for 30 min at 37 °C. Reagents were dried under nitrogen and the residue was reconstituted in 30 μl chloroform and 20 μl methanol and chromatographed on a silica Thin-layer chromatography (TLC) plate to 13 cm in a solvent system of chloroform: methanol (93:7, v/v). The R_f_ of PGF2a methyl ester in this solvent system was 0.15. Compounds migrating in the region 1 cm below the PGF2a standard to 1.0 cm above the standard were scraped from the TLC plate, extracted with 1 ml ethyl acetate, and dried under nitrogen. Following TLC purification, compounds were converted to trimethylsilyl (TMS) ether derivatives by addition of 20 μl N*,O*-bis(trimethylsilyl)trifluoroacetamide, and 10 μl dimethylformamide. The sample was incubated at 37 °C for 10 min and then dried under nitrogen. The residue was re-dissolved for GC/MS analysis in 20 μl undecane that has been stored over a bed of calcium hydride.

### Immunoblotting and western blot analysis

Immunoprecipitation and Western blot analysis were performed as previously described (Sayeski et al. 1998). To determine the association of p47phox with gp91phox, protein isolated from PBMC homogenates of patients treated with placebo and galantamine were immunoprecipitated by adding the gp91phox (antibody (2 μL per sample; Abcam, Cambridge, MA, Cat# ab80508) and Protein G Agarose beads (40 μl per sample; ThermoFisher Scientific, Waltham, MA, Cat# 20398) and incubated at 4 °C overnight. After microcentrifugation and washing the pellet to reduce non-specific binding and remove excess anti-gp91phox antibody, an equal volume of each denatured immunoprecipitation sample was loaded onto a SDS-PAGE gel (12–15%). Western blotting was performed using the primary antibodies rabbit anti-p47phox (1:1000; Millipore, Billerica, MA, Cat# 07-001) and rabbit anti-gp91phox (1:1000; Abcam, Cambridge, MA, Cat# ab80508) followed by incubation with goat anti-rabbit horseradish peroxidase-labeled IgG (1:2500; Invitrogen, Rockford, IL, Cat# 62-9520). Next, we analyzed the ratio of p47phox to gp91phox by quantifying relative protein expression to determine NADPH oxidase formation in these cells.

### Plasma acetylcholine measurements

Twenty microliters of plasma were diluted with acetonitrile: water (80:20, v/v) containing 5% formic acid. The sample was vortexed for 15 s and protein was removed by centrifugation at 10,000*g* for two minutes. Samples were prepared for mass spectrometry as follows: 5 uL of supernatant was added to a 1.5 mL microcentrifuge tube. To that was added 10 uL each of 500 mM NaCO3 (aq) and 2% BZC in acetonitrile. After two minutes, 20 uL of isotopically-labeled internal stand solution (acetylcholine-d9 in 20% acetonitrile containing 3% sulfuric acid) was added to the tube. The solution was transferred to an LC/MS vial for analysis.

Chromatographic separation was performed on a 2.0 × 50 mm, 1.7 µm particle Acquity BEH C18 column (Waters Corporation, Milford, MA, USA) using a Waters Acquity I-Class UPLC with Sample Organizer. Mobile phase A was 15% aqueous formic acid and mobile phase B was acetonitrile. Samples were separated by a gradient of 98–5% of mobile phase A over 6 min at a flow rate of 450 µl/min prior to delivery to a Waters Xevo TQ-S micro triple quadrupole mass spectrometer. Acetylcholine was monitored using a transition of m/z 146 à m/z 87 (retention time = 0.69 min). Acetylcholine-d9 was monitored using a transition of m/z 155 à m/z 87 (retention time = 0.70 min).

### Contrast-enhanced ultrasonography

Contrast-enriched images were acquired in the contralateral forearm (brachioradialis muscle) using a linear-array transducer connected to an ultrasound (L9-3 mm transducer, iU22; Phillips). This equipment allowed real-time imaging using low (0.08) and high (1.2) mechanical index as the contrast (microbubbles; Definity; Bristol-Myers Squibb) was infused at a rate of 1.5 ml/min through the Intravenous (IV) access for 10 min. At steady state (~ 4 min) the high mechanical index (1.2) used destroyed the microbubbles at the start of video recording. Switching to the low index (0.08) made the microbubbles resonate allowing real-time recording of vascular replenishment. Local temperature was measured using a laser Non-Contact Infrared Skin Thermometer. Data was analyzed in our lab using QLAB ultrasound cardiac.

### Sample size and power calculation

The sample size calculation was performed to detect a 30% difference in D F_2_-IsoPs (primary endpoint) in the same study subjects between placebo versus galantamine treatment. Lopes et al. (Lopes et al. 2003) reported that the mean and SD of D F_2_-IsoPs post lipid infusion was 12.0 ± 2.60 pg/mL in AAs. A sample size of 12 AA women had 85% power to detect a difference of 30% in D F_2_-IsoPs between treatments in the same individual. We enrolled a total of 14 AA women to account for attrition and loss of follow-up.

### Statistics

Standard graphing and screening techniques were used to detect outliers and to ensure data accuracy. The data was assessed for normality. If normality was violated, we applied non-parametric analysis methods. Data were analyzed using R version 3.5.3 software and expressed as mean ± SD throughout the manuscript unless otherwise indicated. Summary statistics for both continuous and categorical variables were provided by randomization groups to describe the study sample. We calculated within-subject mean differences and 95% confidence intervals for galantamine versus placebo comparison within each racial group and tested for treatment effect using the paired t-test or sign rank test as appropriate. *P* less than 0.05 was considered statistically significant.

## Results

### Study subjects

**Fig. 2:**
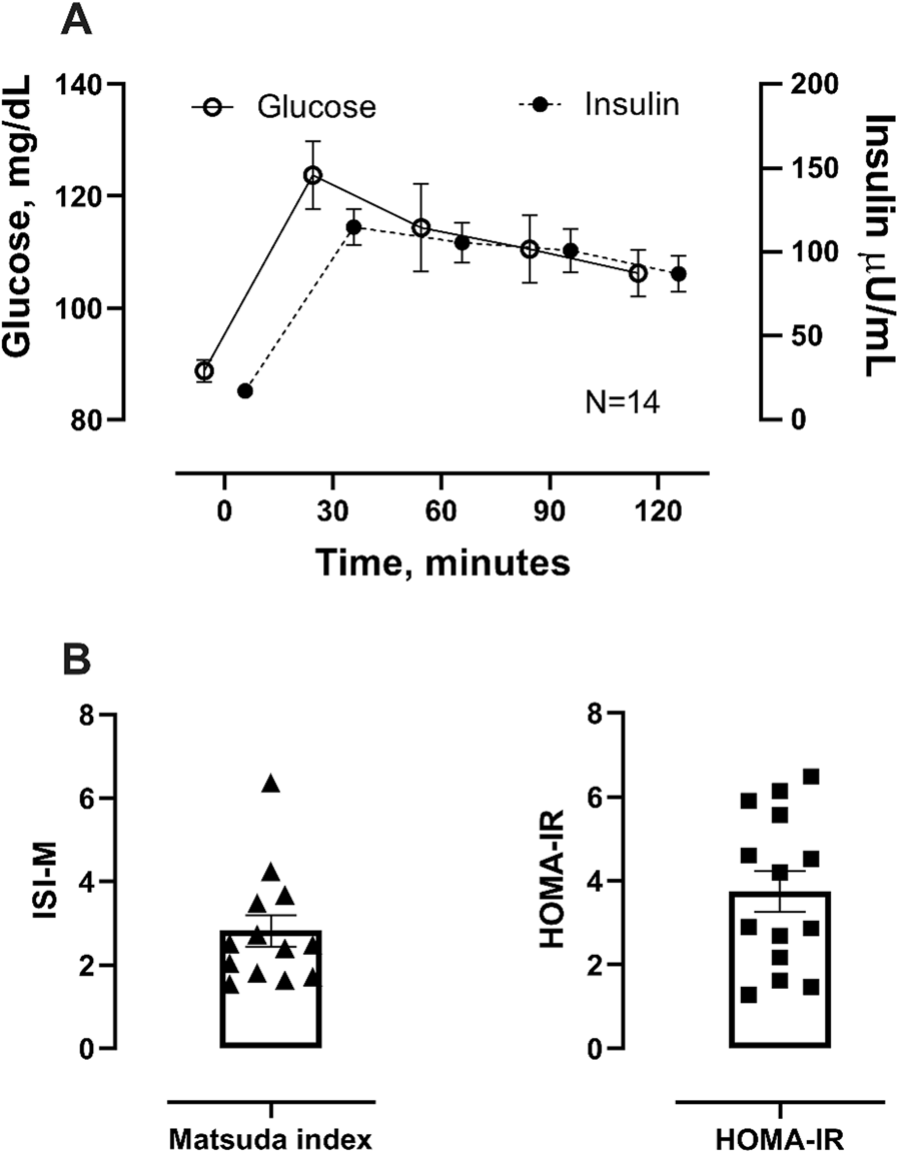
Glucose and insulin excursion during an oral glucose tolerance test. Upper panel (**A**) showed the changes in glucose (Y-left axis) and insulin (Y-right axis) during the 75 g oral glucose tolerance test. Lowe panel (**B**) showed the insulin sensitivity index as calculated by the Matsuda index or HOMA-IR

A total of 32 obese AA women were screened, 18 subjects were excluded because they met exclusion criteria, or withdrew consent. Fourteen subjects were randomized and completed the two study days**,** see Fig. 1, enrollment flow. The baseline characteristics of these patients and the medications they used are presented in the Table 1. Three subjects were on oral contraceptive medications, and two were on inhaled steroids. None of the patients were diagnosed with type 2 diabetes mellitus as per oral glucose tolerance test; 36% of the patients were insulin resistant based on Matsuda index (ISI-M < 3), Fig. 2.

**Table 1:** Demographic characteristics of the study subjects

Age, years	40 ± 10.7
Height, cm	164 ± 5.6
Weight, kg	104 ± 9.8
BMI, kg/m^2^	39 ± 3.4
Free-fat mass, kg	53 ± 4.4
Fat mass, %	51 ± 2.7
Glucose, mg/dl	83 ± 7.5
Insulin, mU/ml	18 ± 7.6
Total cholesterol, mg/dl	165 ± 31.0
HDL, mg/dl	52 ± 12.1
LDL, mg/dl	97 ± 23.0
Triglycerides, mg/dl	85 ± 33.0
Waist size, cm	111 ± 16.0
Systolic BP, mm Hg	121 ± 9.7
Diastolic BP, mm Hg	72 ± 8.2
Heart rate, bpm	67 ± 7.7
Creatinine, mg/dl	0.82 ± 0.07
BUN, mg/dl	12 ± 3.1
*Medications*	
Oral contraceptives	3
Multivitamins	3
Fluticasone inhaler	2

Data presented as mean ± standard deviation of the mean; *BMI* body mass index, *HDL* high density lipoprotein, *LDL* low density lipoprotein, *BP* blood pressure, *BUN* blood urea nitrogen

### Changes in triglycerides and free fatty acids during 20% intralipids® infusion

**Fig. 3:**
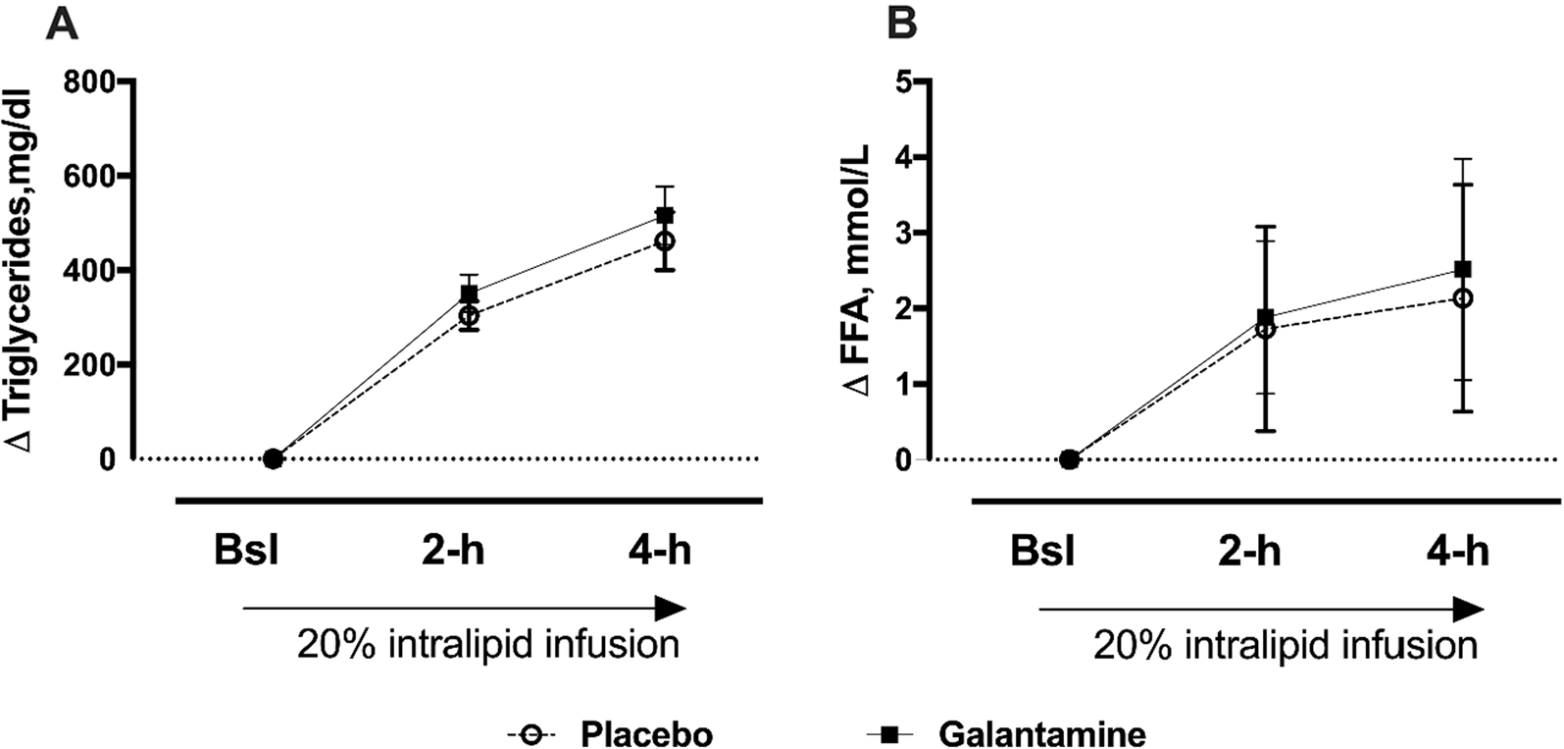
Triglyceride and free fatty acid levels after 20% intralipids® infusion. Change in triglycerides (TGs) and free fatty acids (FFAs) levels during 20% intralipid infusion in the placebo and 16 mg galantamine groups. **A** Serum TGs, **B** Serum FFA. Values shown as means ± SEM (N = 14)

In the same subjects, the increase in triglyceride levels during lipid infusion was similar between placebo and 16 mg of galantamine at 2-h (303.5 ± 108.90 vs. 351.3 ± 143.39 mg/dl, P = 0.340) and 4-h (461.7 ± 221.15 vs. 516.1 ± 222.49, P = 0.685), Fig. 3A. The increase in free fatty acids (FFA) during lipid infusion was also similar between placebo and galantamine at 2-h (1.7 ± 1.35 vs. 1.8 ± 1.01 mmol/l, P = 0.529) and 4-h (2.14 ± 1.50 vs. 2.5 ± 1.46 mmol/l, P = 0.376), Fig. 3B.

### Effect of galantamine on lipid-induced oxidative stress and inflammation

**Fig. 4:**
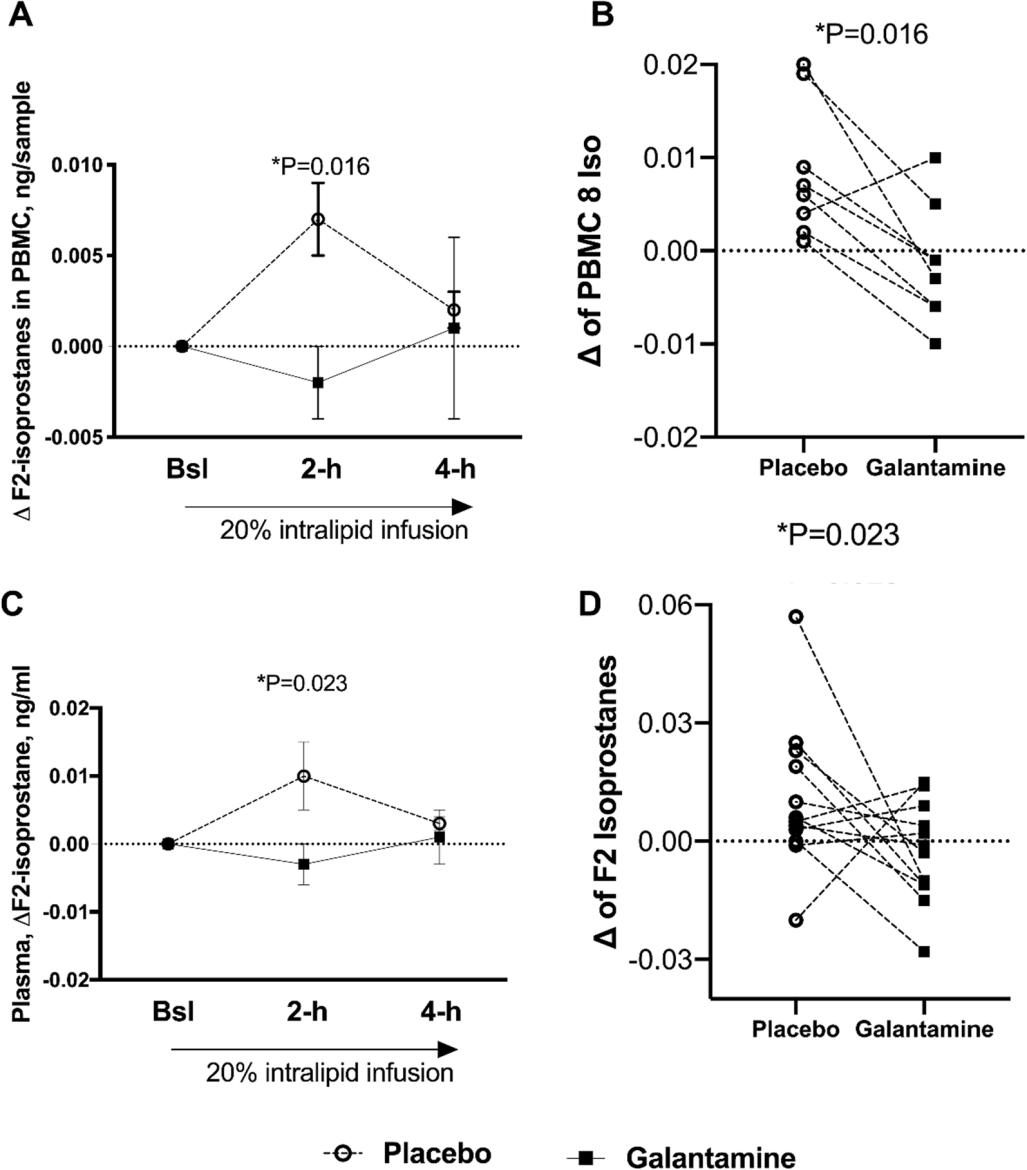
Decreased lipid-induced oxidative stress as measured by F_2_-isoprostances (F_2_-IsoPs) in PBMC and plasma. Baseline-corrected changes in F_2_-IsoPs in PBMC with 16 mg galantamine vs. placebo during 20% intralipid infusion (**A**). Scatterplot showing F_2_-IsoPs individual values in PBMC after placebo and galantamine at 2-h during lipid infusion (**B**). Baseline-corrected changes in circulating F_2_-IsoPs in plasma with galantamine vs. placebo during 20% intralipid infusion (**C**). Scatterplot showing Fs-Iso individual values in plasma after placebo and galantamine at 2-h during lipid infusion (**D**). Values shown as means ± SEM, *P ≤ 0.05 by paired, two-tailed Student *t-*test

Oral administration of 16 mg of galantamine prior to lipid infusion significantly inhibited the increase in F_2_-IsoPs in the PBMC membrane at 2-h (0.007 ± 0.008 vs. − 0.002 ± 0.006 ng/sample, P = 0.016), but not at 4-h (0.002 ± 0.005 vs. 0.001 ± 0.016 ng/sample, P = 0.313), Fig. 4A, B. Galantamine significantly reduced the circulating plasma concentration of F_2_-IsoPs at 2-h (0.01 ± 0.02 vs. − 0.003 ± 0.01 ng/mL with galantamine, P = 0.023) but not at 4-h (0.003 ± 0.01 vs. 0.001 ± 0.02 ng/ml, P = 0.569), Fig. 4C, D. Urine F_2_-IsoPs, normalized to creatinine levels, increased 4-h after lipid infusion, but values were not different between treatments (1.6 ± 1.87 vs. 1.7 ± 1.85 nmol/ml, P = 0.47).

**Fig. 5:**
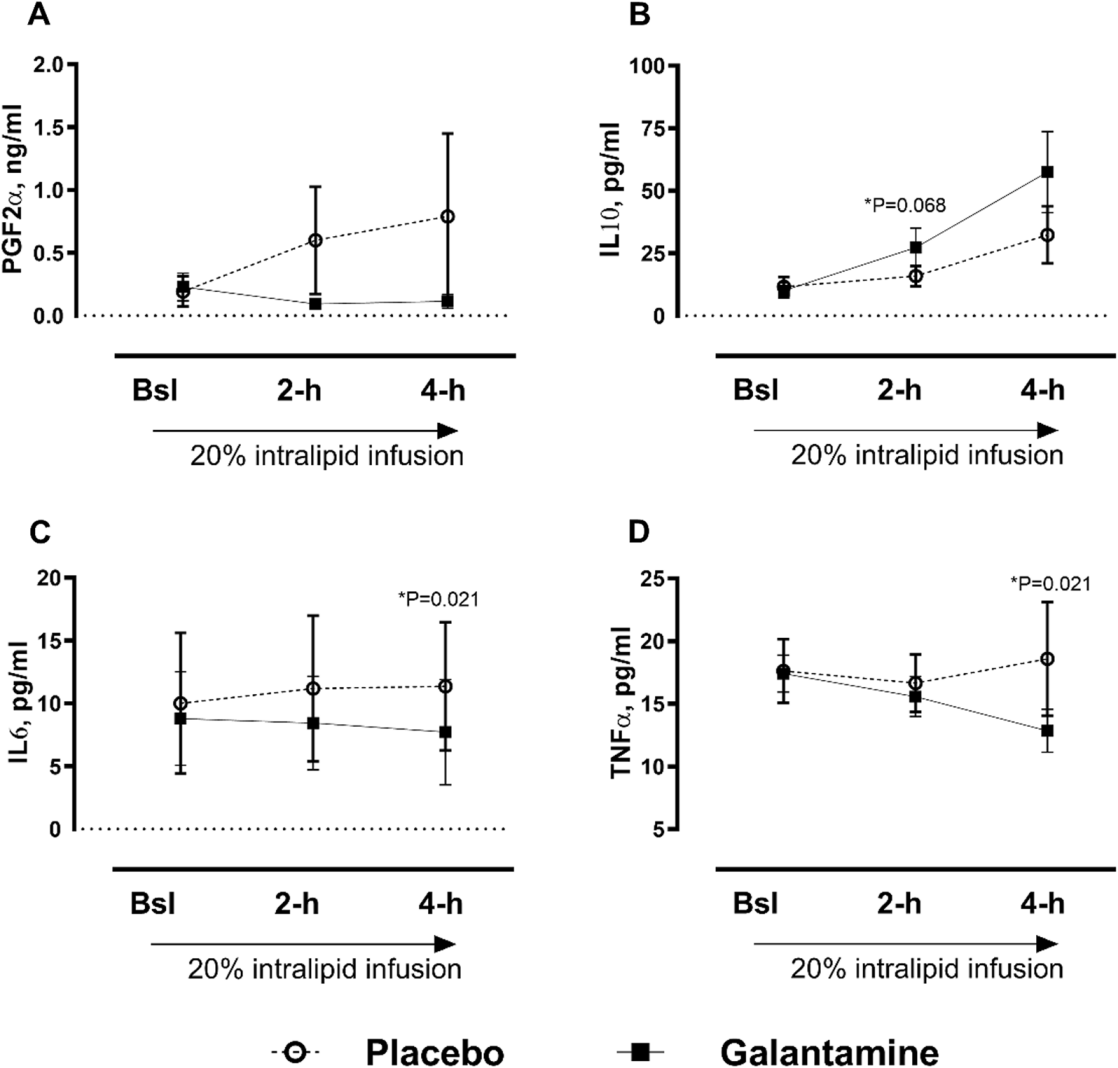
Effect of galantamine on plasma PGF2α and inflammatory cytokines. Change in cytokine concentrations during 20% intralipid infusion in placebo (open circles) and 16 mg galantamine groups (squares). **A** PGF2α serum levels, **B** IL10 serum levels), **C** IL6 levels, **D** TNFα levels. Values shown as means ± SEM, *P ≤ 0.05 by paired, two-tailed Student *t-*test

Infusion of 20% intralipids increased PGF2α with placebo; nevertheless, galantamine did not significantly reduce PGF2α in plasma, Fig. 5A. Compared with placebo, galantamine tended to increase IL-10 levels, anti-inflammatory cytokines at 2-h (15.8 ± 15.00 vs. 27.3 ± 27.91 pg/mL, P = 0.068) and 4-h post-lipid infusion, Fig. 5B. Further, galantamine significantly decreased inflammatory cytokines, IL-6 (11.4 ± 18.45 vs. 7.7 ± 15.11 pg/mL with galantamine, P = 0.021) and TNFα levels (18.6 ± 16.33 vs. 12.9 ± 6.16 pg/mL with galantamine, P = 0.021), Fig. 5C, D.

**Fig. 6:**
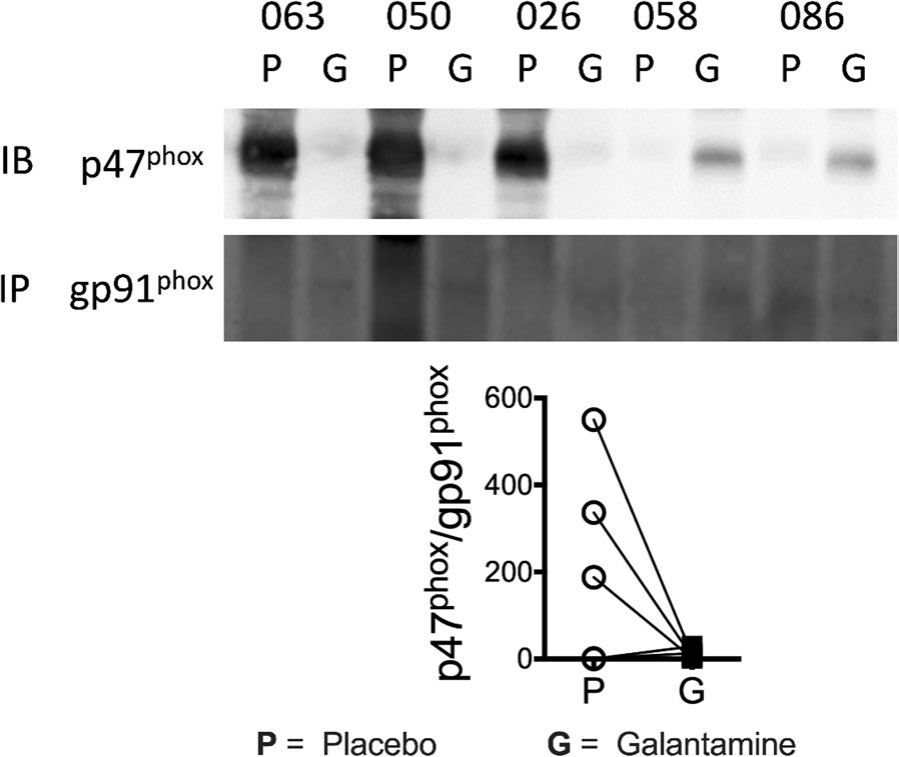
Assessment of NADPH oxidase activation in PBMC with Galantamine. We obtained PBMC in five AAs who showed a robust decline in F_2_-IsoPs in response to galantamine. The NADPH oxidase activity depends on the assembly of the cytosolic subunits p40^phox^, p47^phox^, and p67^phox^ with the membrane-bound subunits p22^phox^ and gp91^phox^. There was not statistically significant difference in NADPH activity with galantamine

Additionally, we examine the effect of galantamine on lipid-induced assembly of NADPH oxidase, we immunoprecipitated gp9^*phox*^ then performed Western blot to detect association with p47^*phox*^. In PBMCs of five AAs, 20% intralipid® infusion with galantamine at 2-h prevented association of p47^*phox*^ with gp91^*phox*^ compared to placebo (215.2 ± 104.80 vs. 11.8 ± 4.90 Relative Intensity), Fig. 6. The data showed a tendency towards a reduction in NADPH oxidase assembly with galantamine treatment.

### Effect of galantamine on microvascular circulation

**Fig. 7:**
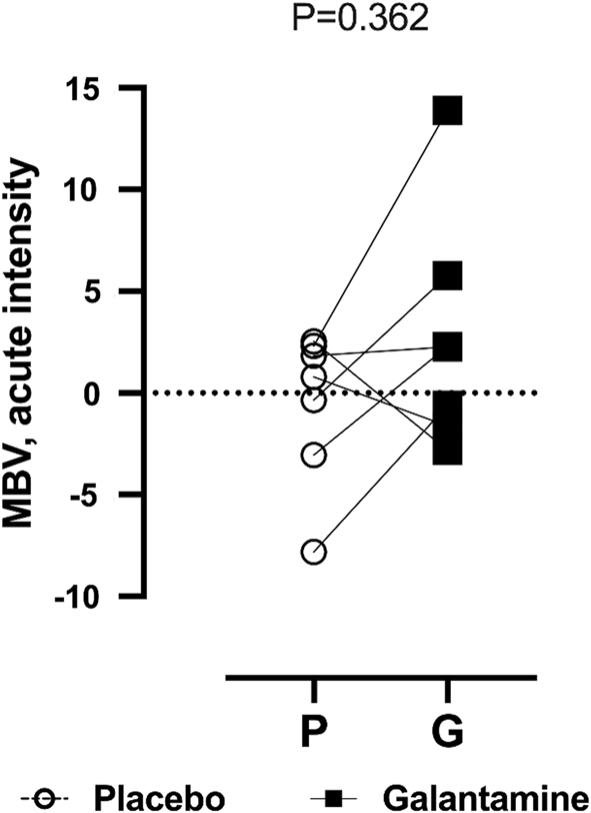
Effect of galantamine on microvascular blood volume in skeletal muscle. Microvascular blood volume (MBV) was measured using contrast enhanced ultrasonography, there were no significant changes in MBV between placebo and galantamine (P = 0.362)

Microvascular circulation was measured at baseline and 4-h after the lipid infusion. Our results did not find any difference in the amount of MBV (microvascular blood volume) between placebo and galantamine during acute hyperlipidemia, (0.19 ± 4.01 vs. 1.25 ± 6.77, P = 0.15), Fig. 7.

### Acetylcholine levels in plasma as biomarker for galantamine’s actions

**Fig. 8:**
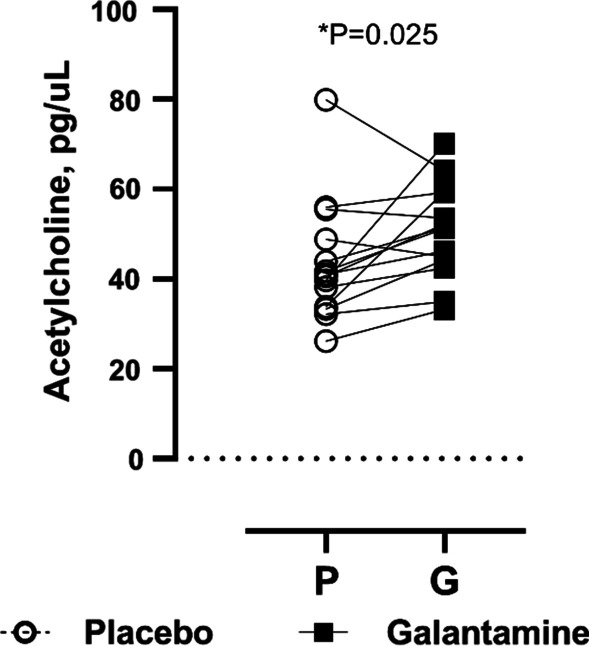
Galantamine increased circulating acetylcholine levels. There was a significant increase (P = 0.025) in acetylcholine levels with galantamine (G) compared with placebo

As expected, galantamine significantly increased acetylcholine levels two hours post-administration (43.6 ± 13.38 vs. 50.5 ± 10.49 pg/uL with galantamine, P = 0.025) which was consistent with the peak effect of the drug, Fig. 8.

### Adverse events

Eight subjects developed mild adverse events (AEs). There were no serious AEs. In the placebo group, two subjects reported nausea and headaches that resolved after the study ended.

In the galantamine group, the most common side effects were severe nausea, headaches, and abdominal cramps.

## Discussion

The main finding of our study was that in obese AA women, galantamine increased PNS cholinergic activity and suppressed lipid-induced oxidative stress as measured by F_2_-IsoPs in PBMC and plasma; this effect was associated with a decrease in the production of inflammatory cytokines, particularly IL-6 and TNFα.

The effect of galantamine on F_2_-IsoPs was evaluated in three different organs (PBMC, plasma, and urine). PBMC is one of the largest sources of ROS; F_2_-IsoPs, an endogenous biomarker of oxidative stress, is produced in vivo by nonenzymatic peroxidation of arachidonic acid esterified in membrane phospholipids, and therefore specifically assess lipid peroxidation. Then, F_2_-IsoPs is released to the circulation by phospholipase A2 activities and excreted in the urine. In AA women, a single dose of galantamine decreased F_2_-IsoPs in PBMC and plasma at 2-h during lipid infusion, which coincided with the drug’s peak effect and shown by the significant increase in Ach levels. Furthermore, at 4-h post-lipid infusion, F_2_-IsoPs in plasma returned to baseline values, but it remained elevated in PBMC. In contrast, we did not observe a significant decline in F_2_-IsoPs in urine collected at 4-h after drug intake, possibly because the maximum effect of galantamine on oxidative stress occurred at 2-h after intake.

Previous studies in animal models found that stimulation of the PNS conferred protection against oxidative stress (Borovikova et al. 2000). Stimulation of the PNS decreased malondialdehyde generated in response to various stimuli including ricin poisoning, myocardial infarction and fulminant hepatitis (Mabley et al. 2009; Abdel-Salam et al. 2013). This effect was in part mediated through the α7nAChR (Hiramoto et al. 2008; Parada et al. 2010) and vagal afferent nerves (Borovikova et al. 2000). Also, sub-diaphragmatic surgical vagotomy in rats exposed to endotoxin was associated with increased oxidative stress in the brain (Egea et al. 2012). Similarly, direct electrical stimulation of the peripheral vagus nerve during lethal endotoxemia inhibited TNF alpha synthesis in the liver (Hiramoto et al. 2008) Altogether, these studies suggest that states of heightened ROS production can be targeted by increasing PNS activity either through direct vagus nerve stimulation or with drugs that increase cholinergic transmission.

In the present study, we selected continuous lipid infusion as stimuli to generate oxidative stress because it is safe to use in humans and AAs had a higher F_2_-IsoPs production than whites (Lopes et al. 2003). This increased susceptibility to heightened oxidative stress production is unknown; previous studies in AA women reported an increased expression of NADPH oxidase, p47^phox^ subunit, NOX2, and NOX4 in baseline conditions (Feairheller et al. 2011). To understand the mechanism underlying the reduction in lipid-induced F_2_-IsoPs production, we evaluated the activity of NADPH oxidase in PBMC of five subjects who showed a robust decline in F_2_-IsoPs in response to galantamine. The NADPH oxidase activity depends on the assembly of the cytosolic subunits p40^phox^, p47^phox^, and p67^phox^ with the membrane-bound subunits p22^phox^ and gp91^phox^. During activation of the NADPH oxidase, p47^phox^ is phosphorylated, and the cytosolic subunits assemble and move to the membrane to form a functional complex with the membrane-bound subunits. Three subjects showed reduction in NADPH oxidase activity with galantamine through a substantial decrease in the association of p47^phox^ with gp91^phox^ during lipid infusion, Fig. 6. Further studies with a large sample size are needed to examine the effect of galantamine on activation of the NADPH oxidase.

The relationship between oxidative stress and inflammation is complex, lipid-induced IL-6 and TNF alpha production, a long side F_2_-IsoPs, significantly decreased with galantamine. It is noteworthy that F_2_-IsoP and isolevuglandins (IsoLGs) work as neo-antigens leading to activation of antigen-presenting cells, T cells, and release of inflammatory cytokines. This autoimmune state has been associated with the development of salt-sensitive hypertension (Kirabo et al. 2014) which disproportionately affect AAs. In future studies, it would be important to determine if galantamine decreases protein-adduct IsoLGs, antigen-presenting cells, and T cell activation.

Finally, our results could have important clinical implications. African Americans, particularly women, have one of the highest prevalence of cardiovascular disease, T2DM, and mortality in the US, which is in part explained by the disproportionate prevalence of cardiovascular risk factors such as hypertension and T2DM. Increased plasma and urinary F_2_-IsoP concentrations have been associated with cardiovascular mortality (Liburd et al. 2005), and oxidative stress and inflammation are factors known to contribute to the pathogenesis of T2DM and hypertension. A previous study showed that Galantamine decreased inflammatory cytokines in an obesity animal model (Satapathy et al. 2011). The fact that galantamine inhibited oxidative stress induced by lipids provided important evidence about the importance of the PNS cholinergic system in AAs; results from this proof-of-concept study could open new therapeutic pathways to treat conditions associated with enhanced oxidative stress in this population. Further, our study also demonstrated that circulating elevated levels of Ach, which increased with galantamine, can be used as a biomarker of galantamine’s action on lipid-induced oxidative stress.

The present study has several limitations; first, the sample was small; however, our strong study design (crossover study, which decreases interindividual variability) and the use of lipid infusion to enrich ROS production, allowed us to reach meaningful conclusions. A larger study would be required to determine the beneficial effect of Galantamine on clinical outcomes in AA. We did not measure other oxidative stress processes like protein and DNA damage derived from oxidation damage, which were shown to be reduced with galantamine in subjects with metabolic syndrome (Sangaleti et al. 2021). Nevertheless, our study focused on F_2_-IsoPs measurements in different system, which is a reliable biomarker of endogenous lipid peroxidation. Lastly, the exact mechanism by which increased acetylcholine levels decreased F_2_-IsoPs is still unknown.

## Conclusions

In summary, our study found that galantamine inhibited lipid-induced oxidative stress as measured by F_2_-IsoPs in PBMC and plasma in AAs; this effect was associated with decreased production of inflammatory cytokines IL-6 and TNF alpha.

## Data Availability

The data support the finding of this study are available in [Vivli] at [https://vivli.org], reference number [NCT02365285]. These data were derived from the following resources available in the public domain: [https://clinicaltrials.gov/ct2/home].

## Abbreviations

AchE: Acetylcholinesterase
AEs: Adverse events
AA: African Americans
BMI: Body mass index
CVD: Cardiovascular disease
CYP: Cytochrome P450
CRC: Clinical Research Center
CEU: Contrast-enhanced ultrasonography
EDTA: Ethylenediaminetetraacetic acid
ECG: Electrocardiogram
F_2_-IsoPs: F_2_-isoprostane
GFR: Glomerular filtration rate
hCG: Human Chorionic Gonadotropin
IL-6: Interleukin 6
IV: Intravenous
IsoLGs: Isolevuglandin
NADPH: Nicotinamide adenine dinucleotide phosphate
NEFA: Non-esterified free fatty acids
PNS: Parasympathetic nervous system
PBMC: Peripheral blood mononuclear cells
PGF2a: Prostaglandin F2a
ROS: Reactive oxygen species
TLC: Thin-layer chromatography
TMS: Trimethylsilyl
TNFa: Tumor necrosis factor
T2DM: Type 2 diabetes mellitus
